# Cancer coverage: the public face of childhood leukaemia in 1960s Britain

**DOI:** 10.1016/j.endeavour.2008.01.004

**Published:** 2008-03

**Authors:** Emm Barnes

**Affiliations:** Wellcome Unit for the History of Medicine, University of Manchester, Manchester M13 9PL, UK

## Abstract

In the 1960s, stories of children fighting cancer, previously absent from the British news, started to feature ever more prominently in the national press. Conventional treatments could not keep children alive for many months, so the promise of a cure through the use of an alternative anti-cancer ‘serum’ was not easily dismissed as quackery. The Ministry of Health and cancer research organisations struggled to find a fair and honest way to inform the public and affected families about childhood leukaemia without raising or crushing hope.

## Introduction

Shortly before Christmas 1963, the British public was introduced to Edward Burke, a little boy from Blackpool suffering from acute leukaemia. His only chance of life, it seemed, was to travel overseas to receive an unorthodox but promising treatment. The major British daily papers followed his progress closely for four months, the first time that a particular child suffering from cancer made the national news. Leading cancer experts, senior officials at the Medical Research Council (MRC), the Ministry of Health, parliament and even the Queen found themselves drawn into Edward's case. His plight exposes changes in journalistic practice, public interest in and understanding of cancer at this time and illustrates the importance of a small, patient-led charity in changing the perception of childhood cancer in Britain forever.

In the 1960s, acute leukaemia was the most common and dreaded of all the cancers affecting children, feared because it was invariably and usually rapidly fatal. While the American media had carried stories about families touched by childhood cancer since at least the 1930s [1], their British counterparts had been virtually silent on the subject until the early 1960s. Then, from 1961 onwards, children with cancer began to appear in the news with increasing frequency, both as a patient category and, with Edward, as individuals.

The sudden entry of such stories into the British press cannot simply be explained by changes in the nature of science journalism in the 1960s: the absence of all mention of children with cancer until this period was deliberate and negotiated. What catapulted paediatric cancer into the spotlight at this time was a battle between medical institutions over the ‘right’ way to talk to the public about such a serious illness and the perceived failure of orthodox medicine to meet the needs of those affected.

## Edward's tale

**Figure 1:**
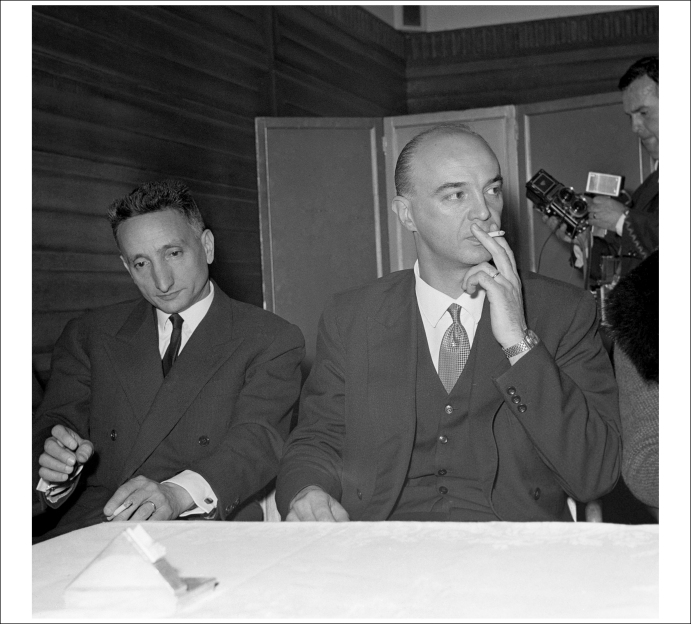
Maverik chemist Gaston Naessens in January 1964, waiting to board an aeroplane to take him from London to the continent. Reproduced, with permission, from PA Photos.

In the latter part of 1963, doctors in Edward Burke's hometown of Blackpool diagnosed him with acute leukaemia but could offer no treatment. Edward's parents, meanwhile, had heard of Gaston Naessens, a French biologist claiming to have a cure for all manner of cancers (Figure 1). Although the French medical authorities had serious concerns about his credentials – he had no recognised diploma in science and had faced trial for practicing medicine without a licence [2] – the Burke family began raising money to transfer their son to France and into his care.

Naessens named his treatment ‘Anablast’ (meaning ‘without cancer cells’), an antibody-based serum produced by immunizing a horse with cells from a patient suffering from cancer. When Naessens accepted Edward as a patient, the chemist came under scrutiny like never before as French government laboratories prepared to put Anablast to the test.

Until it had investigated the safety and efficacy of this treatment, the government announced that no doctor should administer it to their patients, and as Naessens had refused to pay for such tests, he had relocated to Corsica where French law held less authority and was harder to apply. Even then, unwilling to face trial again should he administer the serum himself, he struggled to find a doctor prepared to work alongside him. William Snook, the British Vice-Consul in Corsica who had arranged for Edward to fly out with his mother on 22 December 1963, soon announced that unless a doctor could be found quickly the pair would have to return to the United Kingdom to avoid Edward's health deteriorating further.

**Figure 2:**
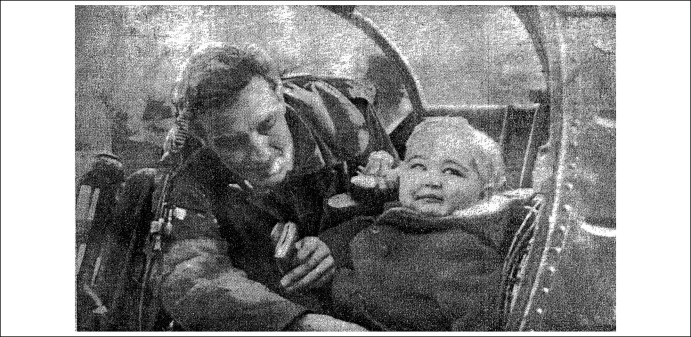
Edward Burke being strapped into a helicopter on 30 December 1963. Reproduced, with permission, from the Daily Telegraph.

That evening, 3000 Corsicans staged a rally in Bastia where the boy was staying to show their sympathy for Edward's plight, bearing placards with slogans that read ‘Quickly, leukaemia does not wait’, and ‘We must save Eddie’. The French government grew alarmed at the scale of the publicity Naessens was attracting and issued a statement: the serum *must* not be given in Corsica either. Any doctor breaking the ban would have their licence revoked, they warned. Nevertheless, a prominent local doctor, Henri Santonacci, announced he was prepared to defy the authorities and administer the serum. On 30 December, *The Daily Telegraph* carried a photograph of Edward being strapped into a helicopter to be flown from Bastia to Ajaccio for his first shots [3] (Figure 2).

In the UK, staff at the Ministry of Health quickly realised they would need to plan for an influx of enquiries from families in a similar situation. ‘It would appear that requests for treatment could well snowball’, warned the Blackpool hospital committee and suggested that the Ministry ascertain the efficacy of the serum as soon as possible to prevent a wave of desperate parents taking children overseas [4].

It was not long before the enquires began, with families writing to Snook, their Councillors, Members of Parliament and the Ministry of Health. The situation threatened to get out of hand. The Ministry, usually determinedly silent on claimed cancer ‘cures’, realised it had to act to prevent scores of families flying to Corsica only to be denied treatment. *The Daily Mail* noted that more than 100 British families had requested treatment with Anablast, but Naessens only had enough serum to treat his existing patients [5]. All this coverage, noted *New Scientist*, had the effect of focusing the public gaze on acute leukaemia, a ‘peculiarly tragic illness’ that almost exclusively and rapidly struck down the young [6].

Throughout January, letters continued to arrive at the Ministry asking them to make the serum more available to British families. Donald Brown, a young boy from Glasgow, flew to Corsica to be treated alongside Edward and five French children; several other families were eager to join them [7]. The parents of a girl from Orkney appealed to their MP to seek advice from the Ministry, a family from Stevenage persuaded their local council to write to Anthony Barber (the Minister of Health) on behalf of their daughter, and a boy from Liverpool was in danger of being withdrawn from care at the Alder Hey Children's Hospital so as to begin treatment with Anablast [8]. The Ministry was under increasing pressure to produce an official government response.

On 6 January 1964, Naessens offered his serum to any government willing to test it. Although the prestigious Parisian Institut Gustav-Roussy took up the offer [9], they warned that definitive results would not be available for up to two years. The UK's Ministry of Health continued to receive demands that it perform the tests more quickly. Eventually, owing to a fund set up by a Scottish businessman, a team of Edinburgh scientists began to evaluate Naessens’ serum and his methods. In the middle of January, a medical panel headed by the highly respected Edinburgh radiotherapist Robert McWhirter convened away from the press for two days [10]. They recommended that the French trials would be sufficient, that the claimed clinical results should be backed up by a controlled study and that Naessens should publish his methods, observations and theories immediately. The Ministry of Health made arrangements to relocate Naessens to Britain at least temporarily, which would ensure a steadier supply of Anablast for British families and coincidentally keep the controversial chemist away from French media attention while the authorities in his homeland investigated whether there was sufficient material to bring a case against him [11].

## Quack remedy

Despite the Institut Gustav-Roussy's projected timescale, they released their preliminary findings within weeks: Naessens’ work was riddled with errors; the serum was useless. The microorganisms that Naessens claimed were uniquely found in the blood of leukaemics were nothing of the sort, Pierre Denoix reported to the French Minister of Public Health. They were, in fact, fragments of red blood cells known as myelinic figures that had already been described, photographed and found to occur in the blood of both diseased and non-diseased patients. What is more, microbes supposedly cultured from the blood of his patients were found to be contaminants. Finally, all of those Naessens claimed to have ‘cured’ using Anablast turned out to have received conventional treatment as well, suggesting his success stories were down to the delayed effects of earlier therapy. Denoix's damning conclusion was that Naessens was peddling nonsense, a product that could not possibly have any effect on leukaemia or, for that matter, solid cancers [12]. The French authorities responded quickly, issuing a statement that no doctor, whether on mainland France or in Corsica, was to administer the serum.

In Britain, where emotive pleas continued to flood in from the public, the Ministry of Health could not afford to be so decisive. ‘I implore you to intervene to save my son’, wrote Edward's father in a telegram to Barber after the French announcement. ‘[T]reatment must not be discontinued at this vital stage otherwise all hope is gone’, he wrote [13]. For Patrick Jenkins, the prospective Conservative candidate for Wanstead and Woodford, the serum was nothing more than a ‘quack remedy’. ‘It does seem wicked that an absolutely unqualified man like Naessens should have aroused such wild hopes in desperate hearts’, he wrote to Barber [14]. The Ministry could not afford to dash such hopes but equally could not be seen to promote an unproven treatment over the chemotherapy regimes then available in the larger children's hospitals. A Ministry employee, writing in an internal memo, summed up the dilemma: ‘[W]e are vulnerable either way, whether we refuse or whether we allow this’ [15].

The solution was a compromise. On 28 January, Barber released a memo stating that small quantities of Anablast could be imported without hindrance for the treatment of particular patients and that doctors would not be penalised if they chose to administer it [16]. Edward's mother Mary telegrammed Queen Elizabeth, appealing to their common motherhood and asking for help protecting Naessens from governmental attacks. Other families struggling to cope with the slim chances for children with leukaemia made similar appeals. In March, Naessens himself joined in, appointing a lawyer to explore the legality of producing the serum in the UK [17]. Staff in the Ministry, meanwhile, sought to be as unhelpful as possible to Naessens, though stopped short of blocking the use of Anablast. As one memo put it, ‘we should keep M. Naessens out if we can; we do not want a Corsica in East Kilbride’ [18].

By April, Edward's plight had slipped out of the media spotlight in favour of exciting findings coming from America: high doses of four drugs given in combination had apparently cured four out of seventeen leukaemic children given the experimental cocktail, reported the *Daily Express* [19]. At last, the Ministry had its hands on some concrete evidence that orthodox treatment for leukaemia, which involved administering steroids, anti-metabolites and other chemicals with proven anti-cancer effects, could work. From here on, peddlers of alternative remedies would prove easier to silence and to contain: the medical profession had found a way to give real hope to families of sick children.

Naessens only returned to the headlines in May the following year, when a French court fined him for fraudulent medical practice [20]. By this time, however, he had emigrated to Canada, established a new laboratory and started developing 714-X, a camphor-based treatment for cancer and ‘other immunologically based diseases’ [21]. Once all hope of curing Edward Burke had vanished, the boy was no longer of interest to the newspapers or of concern to the Ministry of Health, whose files make no mention of his death. Nevertheless, the response to the coverage – the letters written to MPs, Ministries and high-profile figures – suggests that the public felt their government could be doing more for children in Edward's situation.

In fact, the British government had been investing heavily in leukaemia research for a decade. In 1954, David Hewitt of the Oxford Department of Social Medicine prepared a report for the Medical Research Council (MRC) on the changing incidence of serious disease in the United Kingdom [22]. While heart disease and lung cancer were both on the increase, childhood leukaemia was responsible for the greatest increase in years of life lost. Hewitt's report prompted the MRC to reassess its funding priorities and coordinate research into the causes of and treatments for childhood leukaemia [23].

In 1959, the MRC launched its first clinical trials for this disease, but this did not produce any good news about the war on cancer: less than 10% of children diagnosed with the condition were receiving any chemotherapy and of those that were none survived more than 12 months. Consequently, the MRC decided to remain silent about this initiative rather than reveal that government funds were making no appreciable difference to survival and children were being put through unnecessary suffering in their last few months.

## Charitable contributions

The MRC was not the only source of funds for research into childhood cancer: the British Empire Cancer Campaign (BECC) and the Imperial Cancer Research Fund (ICRF) were each also making grants to laboratories, furthering work into the basic biology of cancer and testing possible anti-cancer agents. But, with their funding priorities and outlook similar to those of the MRC, these charities also kept a low media profile. This was, partly, to avoid being seen to favour particular clinicians or treatment centres over others, but also because, like the MRC, they felt it ill-advised to broadcast the slow progress in the fight to conquer cancer.

**Figure 3:**
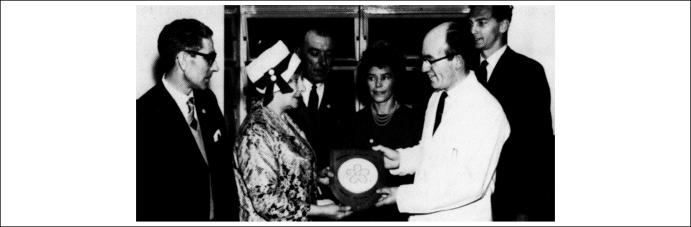
The opening of the Leukaemia Research Unit in December 1961 at the Hospital for Sick Children in Great Ormond Street. Gordon Piller watches on from the far right as Middlesborough Councillor Mrs E Gaunt hands over a plaque to hospital staff on behalf of representatives of the The Teeside Leukaemia Fund (centre). Reproduced, with permission, from the Leukaemia Research Fund.

This silence was shattered by the appearance of the Leukaemia Research Fund. Following the loss of their daughter to acute leukaemia in 1960, two parents founded the charity in their home region of Teeside with designs on establishing an office in London. At the end of 1961, the charity used its first £5000 to establish a leukaemia research unit at the Hospital for Sick Children in Great Ormond Street, the first such facility in a children's hospital in the UK [24] (Figure 3). They put out a press release to celebrate the opening, a story that received near-blanket coverage in the national press. On 7 December, for example, *The Times* carried a short piece about the new unit, welcoming this boost to the hospital's long-standing commitment for fighting childhood leukaemia [25].

The stories about this new charity unleashed a flood of public enquiries to the MRC from relatives of children with the disease and from members of the public wishing to raise funds. Mothers, aunts and grandmothers of sick children wanted to know how to contact the LRF, whether the LRF's unit was developing treatments not available elsewhere, and why it had taken a charity to fund this facility rather than the British government. Throughout the mid-1960s, this last question continued to be asked in daily broadsheets and in questions to parliament: was it right that charitable funds should be needed to pay for research into a major child killer?

Gordon Piller, the first and exceptionally long-standing director of the LRF, was an experienced medical administrator and directed the charity away from the British tradition of reporting only on laboratory research that was being funded (Figure 3). Instead, he imported American models of press and public relations, writing press releases for distribution across the country to capitalize on every mention of childhood cancer. As stories emerged in Uganda about Denis Burkitt's work on a striking form of endemic lymphoma and in the United States about the progress of state-funded research teams seeking maximally effective combinations of chemotherapeutic agents, Piller made sure the LRF had something to say and hope to offer. His social entrepreneurship arguably changed the tradition of charity fundraising initiatives across all the medical fields.

In late March 1964, Piller wrote an article about the state of leukaemia research, versions of which appeared in several local newspapers [26]. ‘[C]onstant and exhaustive research is being continued on a wide front’, he wrote in an introductory paragraph to his article in the *Sheffield Telegraph*. ‘Some of that work is being done by the hon. Secretary of the Leukaemia Research Fund Ltd.’

Piller went on to outline where research was taking place and what it had revealed about the nature of the disease, but also warned that though chemicals could temporarily slow the progression of disease there was no known treatment to which it did not become resistant. He did not hold much faith in Anablast: ‘Claims of cures are reported from time to time from all parts of the world. These produce hope, but seldom add anything to what is already known of this disease’, he wrote. His article closed by suggesting that a sturdier form of hope, a grounded hope, lay instead in supporting conventional research into the disease: ‘a great deal of research is going on and will go on until a cure is found. In the past 20 years our knowledge has considerably advanced and everyone is now waiting for a breakthrough’.

This bold optimism was not to the MRC's liking. Such statements had a tendency to elicit questions in the House of Commons and letters from relatives of the sick about *when* this breakthrough would come. Piller's tendency to promote his charity and its generosity also frustrated MRC staff. In all Piller's many articles, he naturally described the work of his own unit first, but in a manner which MRC staff felt underplayed their contribution to the field: ‘The Leukaemia Research Fund, a national charity for research into the disease, is supporting research at Great Ormond Street Children's Hospital, Derbyshire Royal Infirmary and at Newcastle University. The Medical Research Council is also supporting research of various kinds as are some other charitable bodies’ [27]. In contrast to the MRC and the larger cancer charities, the LRF actively courted publicity. It did, after all, depend on individuals’ donations, and many of its keenest supporters had lost child or adult relatives to leukaemia and wished to keep the disease in the public eye and on the government agenda.

Then, in April 1964, a team at the US National Cancer Institute's clinical centre in Bethesda, Maryland announced that they had developed a multi-drug regime for acute leukaemia that seemed capable of killing every leukaemic cell in a small proportion of their human patients. *The Lancet* celebrated this breakthrough and cancer specialists knew that chemotherapy had the potential to cure acute leukaemia rather than just leaving them in the limbo of remission. From then onwards, those like Naessens seeking publicity for unorthodox treatments found it harder to find sympathetic journalists.

## Conclusion

News stories about children with cancer in the 1960s capitalized on the vulnerability of the victims. The lack of effective therapies, which had heartbreaking and financially ruinous consequences for those affected, made compelling copy. The promise of unorthodox remedies like Anablast raised hope and simultaneously cast doubt on the ability of the medical establishment to produce results. With national dailies appointing full-time correspondents to cover science, technology and medicine for the first time [28], medical innovations began to receive more column inches as journalists attempted to unpick the economic, moral and political implications of an increasing number of dramatic new treatment options. The story of Edward Burke proved opportunity too good to miss.
